# Pharmacokinetics, biological effects, and distribution of (1→3)-β-D-glucan in blood and organs in rabbits

**DOI:** 10.1080/09629359791622

**Published:** 1997-11

**Authors:** Minoru Yoshida, Robert I. Roth, Carl Grunfeld, Kenneth R. Feingold, Jack Levin

**Affiliations:** 1 Fourth Depar tmentof Internal Medicine Teikyo University School of Medicine 74 Mizonokuchi Takatsu-ku Kawasaki City 213 Japan; 2 Department of Laboratory Medicine Metabolism Section University of California School of Medicine the Veterans Administration Medical Center San Francisco CA 94121 USA; 3 Department of Medicine Metabolism Section University of California School of Medicine the Veterans Administration Medical Center San Francisco CA 94121 USA

## Abstract

The pharmacokinetics, biological effects and distribution in blood and organs of 125I-labeled (1→3)-β-D-glucan purified from Candida albicans were analyzed in rabbits during the 24-h period following an intravenous administration.The intravascular half-life of (1→3)-β- D-glucan was 1.8 min in the low-dose group (9.3 μg/kg) and 1.4 min in the high-dose group (222 μg/kg), and the mean (±SD) total body clearance was 1.12 ± 0.30 and 1.17 ± 0.16 ml/min, respectively. The rabbits remained well and (1→3)-β-D-glucan failed to alter blood cell counts. Less than 3% of the ^125^I-(1→3)-β-D-glucan was initially associated with the cellular compartment, and this value decreased further during the 2-h period following administration (*P* = 0.0001). Over 97% of ^125^I-(1→3)-β-D-glucan was associated with cell-free plasma, and the majority in plasma appeared to be present in the unbound form (not associated with lipoproteins or plasma proteins). The liver contained more than 80% of the ^125^I-(1→3)-β-D-glucan detected in the six major organs analyzed.


Mediators of Inflammation, 6, 279–283 (1997)

### Pharmacokinetics, biological effects, and distribution of (1→3)-β-D-glucan in blood and organs in rabbits

Minoru Yoshida[1,CA], Robert I. Roth[2], Carl Grunfeld[3], Kenneth R. Feingold[3] and Jack Levin[2]

[1]Fourth Department of Internal Medicine, Teikyo University School of Medicine, 74 Mizonokuchi, Takatsu-ku, Kawasaki City 213, Japan, [2]Department of Laboratory Medicine, and [3]Department of Medicine, Metabolism Section, University of California School of Medicine, and the Veterans Administration Medical Center, San Francisco, CA 94121, USA

[CA]Corresponding author
Tel: +81 285 44 2111
Fax: +81 285 44 5258

**The pharmacokinetics, biological effects and distribution in blood and organs of $^{125}$I-labeled (1→3)-β-D-glucan purified from *Candida albicans* were analyzed in rabbits during the 24-h period following an intravenous administration. The intravascular half-life of (1→3)-β-D-glucan was 1.8 min in the low-dose group (9.3 µg/kg) and 1.4 min in the high-dose group (222 µg/kg), and the mean (±SD) total body clearance was 1.12 ± 0.30 and 1.17 ± 0.16 ml/min, respectively. The rabbits remained well and (1→3)-β-D-glucan failed to alter blood cell counts. Less than 3% of the $^{125}$I-(1→3)-β-D-glucan was initially associated with the cellular compartment, and this value decreased further during the 2-h period following administration (*P* = 0.0001). Over 97% of $^{125}$I-(1→3)-β-D-glucan was associated with cell-free plasma, and the majority in plasma appeared to be present in the unbound form (not associated with lipoproteins or plasma proteins). The liver contained more than 80% of the $^{125}$I-(1→3)-β-D-glucan detected in the six major organs analyzed.**

**Keywords:** (1→3)-β-D-glucan, pharmacokinetics, biological effects

### Introduction

Invasive deep mycoses are becoming increasingly common. Recently, a test to determine the plasma concentration of (1→3)-β-D-glucan, a ubiquitous constituent of fungi, has been developed in order to allow an early diagnosis of deep mycosis and fungal sepsis [1,2].

Knowledge of the biological activities of (1→3)-β-D-glucan is expanding, with the recent recognition that this substance can stimulate the production of platelet-activating factor, tumor necrosis factor, interleukin-1 (IL-1), IL-1 receptor antagonist and several prostaglandins in some experimental models [3–5]. The (1→3)-β-D-glucan receptor has been identified in monocytes [6] and also has been found in several other inflammatory cells. Therefore, in the case of deep mycosis or fungemia, fungi could activate cells through the (1→3)-β-D-glucan receptor, and cause inflammatory responses. Also of interest is the metabolic fate of (1→3)-β-D-glucan *in vivo*. In endotoxemia, the majority of lipopolysaccharide is found in cell-free plasma, especially in the high-density lipoprotein (HDL) fraction. This lipopolysaccharide–HDL complex is thought to be less bioactive than free lipopolysaccharide [7]. However, no information is available about potential associations between (1→3)-β-D-glucan and plasma proteins. Therefore we evaluated the pharmacokinetics of intravenously administered (1→3)-β-D-glucan and its distribution between blood cells and plasma proteins, using radiolabeled (1→3)-β-D-glucan purified from *Candida albicans*.

### Methods

### (1→3)-β-D-glucan

(1→3)-β-D-glucan was purified from *Candida albicans* [Institute for Fermentation (IFO) 1385, American Type Culture Collection (ATCC) 18804]. The average molecular weight of glucan dissolved in NaOH was 92 000. Purified glucan was dissolved with 0.09 N NaOH, and radio-iodination with $^{125}$I was then performed using the method initially described for radiolabeling endotoxin [8]. The specific activity was 2.50 µCi/µg. The purified (1→3)-β-D-glucan contained less than 20 pg endotoxin/mg glucan, as determined by the endotoxin-specific chromogenic limulus test (Endospecy, Seikagaku Corporation, Tokyo, Japan). Contamination by endotoxin during radiolabeling was minimal (1.2 ng endotoxin/mg $^{125}$I-(1→3)-β-D-glucan).

### Experimental protocol

New Zealand White female rabbits were divided into three groups: (1) control (distilled water group)

© 1997 Rapid Science Publishers

($n$ = 2), (2) low-dose (1→3)-β-D-glucan ($n$ = 3) and (3) high-dose (1→3)-β-D-glucan ($n$ = 3). A bolus intravenous injection of 9.3 μg/kg $^{125}$I-(1→3)-β-D-glucan (specific activity 2.50 μCi/μg; low-dose group) or a mixture of 12.1 μg/kg $^{125}$I-(1→3)-β-D-glucan plus 209.9 μg/kg non-radiolabeled (1→3)-β-D-glucan (total 222 μg/kg, specific activity 0.14 μCi/μg; high-dose group) was administered into a marginal ear vein. Blood samples for measurement of the intravascular clearance of (1→3)-β-D-glucan were drawn from the opposite ear vein using a capillary tube (70 μl), at 0.25, 0.5, 1, 2, 3, 5, 10, 15, 20, 25, 30, 60 and 90 min, and 2, 3, 6, 12 and 24 h. Radioactivity of the whole blood samples was measured with a γ-counter (model 500; Packard Instrument Co., Downers Grove, Illinois, USA).

Blood samples (3 ml) for blood cell counts and for the fractionation of cellular and plasma components were also drawn at 1, 5, 10 and 30 min, and 2 and 24 h. Blood samples (2 ml) for the Fungitec G test (Seikagaku Corporation) were obtained at 1, 2, 5, 10, 20, 30, 60 and 90 min, and 2, 3, 6, 12 and 24 h. During the 24-h period of observation, the rabbits remained well.

To study the distribution of $^{125}$I-β-glucan in whole blood *in vitro*, blood samples (3 ml) were collected before administration of (1→3)-β-D-glucan. These samples were then incubated with 200 μl radiolabeled (1→3)-β-D-glucan (85.4 ng) for 20 min at room temperature, before fractionation.

### Analysis of $^{125}$I-(1→3)-β-D-glucan clearance in whole blood

To determine the $^{125}$I-(1→3)-β-D-glucan clearance in whole blood, the time 0 value (counts/min per ml whole blood) was calculated using the formula: total counts/min of injected $^{125}$I-(1→3)-β-D-glucan/estimated blood volume (70 ml/kg × rabbit weight in kilograms). The intravascular half-life ($T_{1/2}$) was determined from the clearance curve. The area under the concentration–time curve (AUC in ng/ml × min) was calculated by trapezoid integration: $\Sigma(1/2(t_{n+1} - t_n)(C_{n+1} + C_n)$, where $t$ is time in minutes, $n$ is time point of blood sampling and $C$ is concentration. Total body clearance was then calculated as: total dose of injected $^{125}$I-(1→3)-β-D-glucan/AUC (in ml/min).

### Determination of (1→3)-β-D-glucan concentration in serum using the G test

The (1→3)-β-D-glucan concentration in serum was determined with the G test [9]. (1→3)-β-D-glucan extracted from the mushroom *Poria cocos* was used as a standard. Since the factor G-activating activity of (1→3)-β-D-glucan purified from *Candida albicans* was 1:200 on a weight basis compared to *Poria cocos*, the concentration of (1→3)-β-D-glucan, as measured by the G test, was multiplied by 200 to obtain the corrected concentrations for *Candida albicans*. Serum samples outside the range of the standard curve were measured after dilution with distilled water. The limit of detection in water (or 0.9% NaCl) of *Poria cocos* was 1.0 pg/ml.

### Fractionation of whole blood and separation of lipoproteins from cell-free plasma

Fractionations were performed at room temperature, as described previously [10]. Platelets, mononuclear cells, polymorphonuclear leukocytes, erythrocytes and cell-free plasma were collected. Cell-free plasma was subjected to sequential ultracentrifugation at 8°C at plasma density (1.006 g/ml) for 18 h, at a density of 1.063 g/ml with KBr for 18 h, and finally at a density of 1.21 g/ml with KBr for 40 h in order to isolate very-low-density lipoproteins (VLDL), low-density lipoproteins (LDL) and HDL, respectively, and to isolate lipoprotein-deficient plasma (density >1.21 g/ml). Following *in vitro* incubation of $^{125}$I-(1→3)–β-D-glucan with normal saline (0.9% NaCl), these steps were also performed to determine the density distribution pattern of (1→3)-β-D-glucan.

### Separation of lipoproteins by precipitation

Separation of VLDL and LDL from serum was performed using the method provided by Sigma Diagnostic Kit 352-4 (Sigma Chemicals, St Louis, Missouri, USA). The separation of all lipoproteins (VLDL, LDL and HDL) from serum was performed using dextran and $MgCl_2$.

### Separation of serum proteins by gel-permeation chromatography

Solutions of $^{125}$I-(1→3)–β-D-glucan in water (85.4 ng in 200 μl) and in rabbit serum (after incubation of $^{125}$I-(1→3)–β-D-glucan with 1 ml rabbit serum for 30 min at 37°C *in vitro*) were chromatographed on a Superose 12 column, using a fast protein liquid chromatography system (Pharmacia LKB Biotech, Uppsala, Sweden). Proteins were monitored at $A_{280}$, and $^{125}$I-(1→3)–β-D-glucan was monitored by determination of $^{125}$I in counts/min.

### Distribution of $^{125}$I-(1→3)-β-D-glucan in tissues

The distribution of $^{125}$I-(1→3)–β-D-glucan in liver, spleen, kidney, adrenal, lung and heart was studied in animals killed 24 h after the injection.

### Data analysis

A two-tailed $t$-test and analysis of variance with repeated measures were performed. $P < 0.05$ was considered significant.

**Table 1.** Pharmacokinetics after an intravenous injection of (1→3)-β-D-glucan

| Dose (μg/kg) | AUC (ng/ml × min) | Total body clearance (ml/min) | $T_{1/2}$ (min) |
|---|---|---|---|
| Low dose (9.3) | 11 811 ± 2 718 | 1.12 ± 0.30 | 1.8 |
| High dose (222) | 460 451 ± 75 982 | 1.17 ± 0.16 | 1.4 |

Values are means ± SD. AUC, area under the concentration–time curve; $T_{1/2}$, half-life.

### Results

### Intravascular clearance of $^{125}$I-(1→3)-β-D-glucan

Fig. 1a shows the pattern of intravascular clearance of $^{125}$I-(1→3)-β-D-glucan during the initial 3 h. The intravascular half-life ($T_{1/2}$) of $^{125}$I-(1→3)-β-D-glucan in the low-dose group was 1.8 min, and that in the high-dose group was 1.4 min (not significantly different). The AUC and total body clearance in each group are shown in Table 1.

Rapid clearance of glucan biological activity was demonstrated by the G test for both low- and high-dose groups, with $T_{1/2}$ values of 4.1 and 2.1 min, respectively (Fig. 1b). There was a good correlation between the isotopic and the biologic methods.

### Blood cell levels

The effects of (1→3)-β-D-glucan on blood cell levels were minimal over 24 h (data not shown).

### Distribution of $^{125}$I-(1→3)-β-D-glucan in blood

Almost all $^{125}$I-(1→3)-β-D-glucan was associated with the cell-free plasma in both groups (Table 2). Less than 3% was associated with the cellular compartment, and this value decreased further during the initial 2-h period after $^{125}$I-(1→3)-β-D-glucan administration ($P$ = 0.0001).

### Distribution of $^{125}$I-(1→3)-β-D-glucan in the cellular compartment

Most $^{125}$I-(1→3)-β-D-glucan in blood cells was associated with platelets 1 min after the injection. After 2 h, the percentage associated with platelets decreased ($P$ = 0.0001). Concomitantly, the proportion of $^{125}$I-(1→3)-β-D-glucan associated with polymorphonuclear neutrophils and erythrocytes increased ($P$ = 0.0008 and $P$ = 0.0001, respectively). However, the absolute quantity of $^{125}$I-(1→3)-β-D-glucan associated with both fractions decreased because the percentage in the cellular compartment decreased from 2.5–3.0 to 0.4% during this period (Table 3).

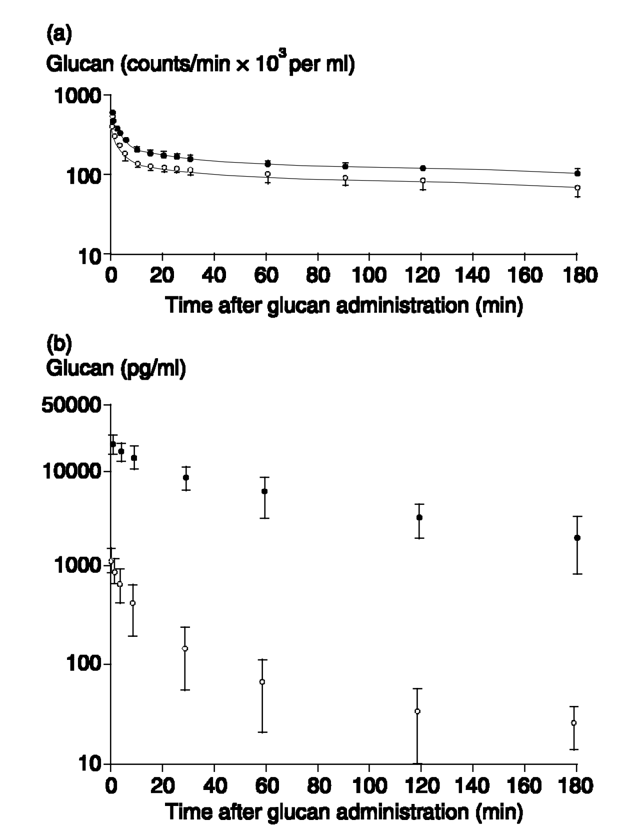

FIG. 1. Intravascular clearance (means ± SEM) of (a) $^{125}$I-(1→3)-β-D-glucan and (b) total (1→3)-β-D-glucan determined by the G test, after intravenous administration in rabbits. Open circles, low-dose group [9.3 μg/kg of $^{125}$I-(1→3)-β-D-glucan, $n$ = 3]; closed circles, high-dose group [12.1 μg/kg of $^{125}$I-(1→3)-β-D-glucan plus 209.9 μg/kg of non-radiolabeled (1→3)-β-D-glucan, $n$ = 3].

### Distribution of $^{125}$I-(1→3)-β-D-glucan among the components of cell-free plasma

More than 60% of the injected $^{125}$I-(1→3)-β-D-glucan was recovered from the lipoprotein-free fraction

**Table 2.** Distribution of $^{125}$I-(1→3)-β-D-glucan (counts/min) in whole blood

| | % Plasma | | % Cells | |
|---|---|---|---|---|
| | Low dose | High dose | Low dose | High dose |
| Time after injection | | | | |
| 1 min | 97.0 ± 0.3 | 97.5 ± 0.8 | 3.0 ± 0.3 | 2.5 ± 0.8 |
| 5 min | 97.7 ± 1.5 | 98.6 ± 0.3 | 2.3 ± 1.5 | 1.4 ± 0.3 |
| 10 min | 98.8 ± 0.5 | 99.1 ± 0.3 | 1.2 ± 0.5 | 0.9 ± 0.2 |
| 30 min | 99.3 ± 0.2 | 99.4 ± 0.2 | 0.7 ± 0.2 | 0.6 ± 0.1 |
| 120 min | 99.6 ± 0.2 | 99.6 ± 0.2 | 0.4 ± 0.2 | 0.4 ± 0.2 |
| *In vitro* incubation | 99.1 ± 0.3 | | 0.9 ± 0.3 | |

Values are means ± SD.

**Table 3.** Distribution of $^{125}$I-(1→3)-β-D-glucan (counts/min) in blood cellular compartments

| | % Platelets | | %MNC | | %PMN | | % Erythrocytes | |
|---|---|---|---|---|---|---|---|---|
| | Low dose | High dose | Low dose | High dose | Low dose | High dose | Low dose | High dose |
| Time after injection | | | | | | | | |
| 1 min | 51.9 ± 8.4 | 70.4 ± 6.1 | 33.4 ± 8.9 | 14.6 ±10.6 | 5.2 ± 2.4 | 6.9 ± 5.4 | 9.5 ± 3.6 | 8.1 ± 2.3 |
| 5 min | 53.4 ± 19.0 | 61.2 ± 11.6 | 23.4 ± 7.8 | 11.6 ±11.8 | 9.3 ± 5.4 | 11.5 ± 6.8 | 13.9 ± 7.2 | 15.8 ± 3.1 |
| 10 min | 48.2 ± 32.0 | 36.7 ± 4.9 | 17.1 ± 17.1 | 16.3 ± 14.4 | 10.2 ± 4.5 | 21.0 ±15.9 | 24.4 ± 10.7 | 26.0 ± 3.6 |
| 30 min | 25.5 ± 7.6 | 22.1 ± 6.2 | 22.3 ±23.3 | 23.8 ± 8.7 | 12.5 ± 3.8 | 20.0 ± 2.4 | 39.7 ± 12.5 | 34.0 ± 3.6 |
| 120 min | 17.9 ± 19.4 | 5.1 ± 8.8 | 0 ± 0 | 19.1 ± 20.5 | 30.7 ± 11.8 | 38.8 ± 23.8 | 51.4 ± 20.0 | 36.9 ± 9.8 |
| *In vitro* incubation | 44.2 ± 10.5 | | 17.3 ± 11.5 | | 14.1 ± 7.9 | | 24.4 ± 11.9 | |

100% represents total counts/min of $^{125}$I-(1→3)-β-D-glucan in the cellular compartment; MNC, mononuclear cells; PMN, polymorphonuclear neutrophils.

**Table 4.** $^{125}$I-(1→3)-β-D-glucan distribution (% total counts/min) in normal saline and cell-free plasma

| | Density <1.006 g/ml | | Density 1.006–1.063 g/ml | | Density 1.063–1.21 g/ml | | Density >1.21 g/ml | |
|---|---|---|---|---|---|---|---|---|
| Sample | Low dose | High dose | Low dose | High dose | Low dose | High dose | Low dose | High dose |
| Normal saline | 13.3 ± 0.4 | | 7.7 ± 1.1 | | 14.7 ± 3.7 | | 64.5 ± 4.6 | |
| Plasma | | | | | | | | |
| *In vitro* incubation | 9.5 ± 0.7 | | 7.2 ± 0.5 | | 17.7 ± 0.9 | | 65.6 ± 2.1 | |
| Time after injection | | | | | | | | |
| 1min | 8.4 ± 1.7 | 9.8 ± 1.0 | 6.8 ± 1.7 | 7.4 ± 0.9 | 16.3 ± 2.5 | 19.1 ± 2.1 | 68.5 ± 5.0 | 63.7 ± 3.6 |
| 5 min | 10.7 ± 1.4 | 11.8 ± 0.5 | 8.1 ± 0.8 | 8.7 ± 0.6 | 20.2 ± 1.3 | 22.1 ± 0.9 | 60.9 ± 3.5 | 57.5 ± 1.5 |
| 10 min | 12.0 ± 0.9 | 12.4 ± 0.5 | 9.2 ± 0.6 | 9.4 ± 0.4 | 21.3 ± 0.3 | 23.0 ± 1.0 | 57.5 ± 1.6 | 55.3 ± 1.7 |
| 30 min | 12.8 ± 0.2 | 13.6 ± 0.5 | 10.0 ± 0.2 | 10.2 ± 0.4 | 23.4 ± 0.4 | 24.4 ± 0.1 | 53.7 ± 0.5 | 51.8 ± 0.8 |
| 120 min | 14.2 ± 0.5 | 14.9 ± 0.4 | 11.3 ± 0.1 | 11.8 ± 0.5 | 25.0 ± 1.1 | 25.0 ± 0.3 | 49.5 ± 0.8 | 48.3 ± 1.0 |

(density >1.21 g/ml). Two hours after the administration of $^{125}$I-(1→3)-β-D-glucan, there was a small but significant increase in the proportion of $^{125}$I-(1→3)-β-D-glucan associated with the three lower-density fractions ($P = 0.0001$ for each fraction), and a significant decrease in the fraction with a density of >1.21 g/ml ($P = 0.0001$; Table 4).

The KBr density centrifugation results suggest that (1→3)-β-D-glucan in blood had only minimal association with lipoproteins. To examine this by independent techniques, solutions of $^{125}$I-(1→3)-β-D-glucan in normal saline and in serum samples obtained 3, 10 and 120 min after administration of $^{125}$I-(1→3)-β-D-glucan were subjected to conditions known to precipitate lipoproteins (one for the precipitation of VLDL and LDL only, and the other for VLDL, LDL and HDL). With precipitation by the former technique, only 4.1% of $^{125}$I-(1→3)-β-D-glucan was recovered in the VLDL + LDL precipitates from serum, and this was no different from the proportion precipitated from normal saline (3.9%). The second technique, for precipitation of all lipoproteins, precipitated 10% of the $^{125}$I-(1→3)-β-D-glucan in serum compared to 1.9% from a saline solution of $^{125}$I-(1→3)-β-D-glucan, suggesting some association with HDL.

In the gel-permeation chromatographic analysis of rabbit serum, all lipoproteins appeared in the void volume, and therefore were distinguishable from other plasma proteins. The patterns of distribution of radioactivity in the solutions of $^{125}$I-(1→3)-β-D-glucan in rabbit serum or in water were almost identical; 47 and 51% were recovered in the void volume fractions, respectively, and the remainder was recovered in the included volume (Fig. 2).

### Distribution of $^{125}$I-(1→3)-β-D-glucan in tissues

The liver contained more than 80% of the $^{125}$I-(1→3)-β-D-glucan distributed in the six major organs, fol-

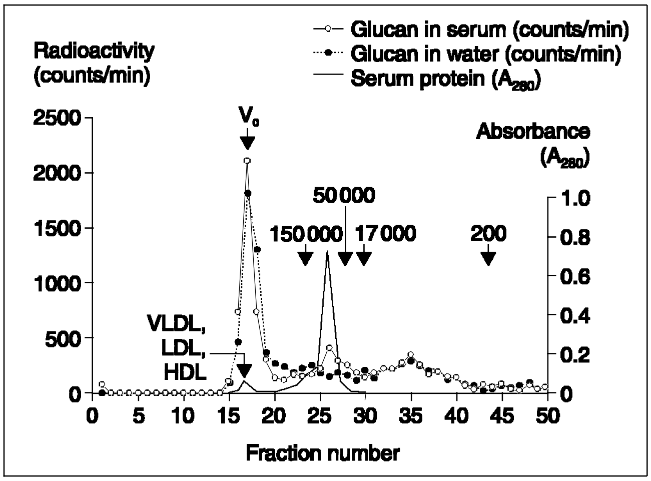

FIG. 2. Separation of rabbit serum proteins by gel-permeation chromatography. Solutions of $^{125}$I-(1→3)-β-D-glucan in water or rabbit serum were chromatographed on a Superose 12 column. Proteins were monitored at $A_{280}$ and (1→3)-β-D-glucan was monitored by determination of $^{125}$I in counts/min. The void volume ($V_0$) and the molecular weight standards are indicated. VLDL, very-low-density lipoprotein; LDL, low-density lipoprotein; HDL, high-density lipoprotein.

lowed by the kidney (approximately 10%). In both dose groups, the spleen, liver and kidney contained high concentrations of $^{125}$I-(1→3)-β-D-glucan.

### Discussion

On the basis of our clinical observations [1,2,11], the elevation of plasma (1→3)-β-D-glucan levels in patients with fungemia is more common and more prolonged than elevated endotoxin levels in Gram-negative bacteremia [12]. However, the intravascular clearance of $^{125}$I-β-D-glucan was unexpectedly rapid. This suggests that a continuous release of β-glucan would be necessary to maintain high plasma levels in patients with fungemia. Despite the reported biological activities of β-glucan, injected soluble (1→3)-β-D-glucan did not produce significant alterations in blood cell levels. Moreover, the administration of (1→3)-β-D-glucan did not produce a significant alteration in the production of tumor necrosis factor (data not shown).

In the present study, almost all injected (1→3)-β-D-glucan was found in cell-free plasma. After sequential ultracentrifugation, more than 60% of injected (1→3)-β-D-glucan was recovered from the lipoprotein-free fraction (density >1.21 g/ml). The VLDL, LDL and HDL fractions were each associated with approximately 10% of the injected (1→3)-β-D-glucan in the early time-point samples. However, a similar density distribution pattern was obtained when the (1→3)-β-D-glucan had been incubated with normal saline *in vitro*. These results indicated that the distribution of (1→3)-β-D-glucan found in plasma fractions does not represent a true binding to lipoproteins or other plasma proteins, but rather is probably a reflection of the heterogeneity of our preparation of (1→3)-β-D-glucan, which contained components with different densities. Our data from gel-permeation chromatography support this interpretation. Approximately 50% of the $^{125}$I-(1→3)-β-D-glucan in either water or serum was recovered from the void volume fraction (which contains all lipoproteins) and the remainder eluted broadly within the included volume ($<2 \times 10^6$ daltons) without any alteration in size distribution, suggesting that binding of $^{125}$I-(1→3)-β-D-glucan to plasma proteins was unlikely. In addition, based on the results of both lipoprotein precipitation methods, only a minor proportion of $^{125}$I-(1→3)-β-D-glucan apparently bound to HDL, and binding to VLDL and LDL was not demonstrated.

In conclusion, soluble (1→3)-β-D-glucan purified from *Candida albicans* showed a rapid clearance rate and failed to alter blood cell counts. Most (1→3)-β-D-glucan seems to occur in the unbound form in plasma. It is unlikely that circulating (1→3)-β-D-glucan is the molecule responsible for the pathophysiologic changes observed in patients with deep mycosis or fungemia.

### References

1. Obayashi T, Yoshida M, Tamura H, Aketagawa J, Tanaka S, Kawai T: Determination of plasma (1→3)-β-D-glucan: a new diagnostic aid to deep mycosis. *J Med Vet Mycol* 1992, **30**:275–280.
2. Obayashi T, Yoshida M, Mori T, *et al.*: Plasma (1→3)-β-D-glucan determination in the diagnosis of invasive deep mycosis and fungal febrile episodes. *Lancet* 1994, **345**:17–20.
3. Abel G, Czop JK: Stimulation of human monocyte β-glucan receptors by particles induces production of TNF-α and IL-1β. *J Immunopharmacol* 1992, **14**:1363–1373.
4. Elstad MR, Parker CJ, Cowley FC, *et al.*: CD11b/CD18 integrin and a β-glucan receptor act in concert to induce the synthesis of platelet-activating factor by monocytes. *J Immunol* 1994, **152**:220–230.
5. Hoffman OA, Standing JE, Limper AH: *Pneumocystis carinii* stimulates tumor necrosis factor-α–release from alveolar macrophages through a β-glucan-mediated mechanism. *J Immunol* 1993, **150**:3932–3940.
6. Czop JK, Kay J: Isolation and characterization of β-glucan receptors on human mononuclear phagocytes. *J Exp Med* 1991, **173**:1511–1520.
7. Ulevitch RJ, Johnston AR, Weinstein DB: New function for high density lipoproteins. Their participation in intravascular reactions of bacterial lipopolysaccharides. *J Clin Invest* 1979, **64**:1516–1524.
8. Ulevitch RJ: The preparation and characterization of a radioiodinated bacterial lipopolysaccharide. *Immunochemistry* 1978, **15**:157–164.
9. Tamura H, Arimoto Y, Tanaka S, Yoshida M, Obayashi T, Kawai T: Automated kinetic assay for endotoxin and (1→3)-β-D-glucan in human blood. *Clin Chim Acta* 1994, **226**:109–112.
10. Yoshida M, Roth RI, Levin J: The effect of cell-free hemoglobin on plasma clearance and cellular, plasma, and organ distribution of bacterial endotoxin in rabbits. *J Lab Clin Med* 1995, **126**:151–160.
11. Iwama A, Yoshida M, Miwa A, Obayashi T, Sakamoto S, Miura Y: Improved survival from fungemia in patients with haematological malignancies: analysis of risk factors for death and usefulness of early antifungal therapy. *Eur J Haematol* 1993, **51**:156–160.
12. Yoshida M, Obayashi T, Tamura H, *et al.*: Diagnostic and prognostic significance of plasma endotoxin determination in febrile patients with haematological malignancies. *Eur J Cancer* 1994, **30**:145–147.
